# Pharmacist Compliance With Therapeutic Guidelines on Diagnosis and Treatment Provision

**DOI:** 10.1001/jamanetworkopen.2019.7168

**Published:** 2019-07-17

**Authors:** Harriet Smith, Stephen Whyte, Ho Fai Chan, Gregory Kyle, Esther T. L. Lau, Lisa M. Nissen, Benno Torgler, Uwe Dulleck

**Affiliations:** 1 School of Economics and Finance, Queensland University of Technology, Brisbane, Queensland, Australia; 2 Centre for Behavioural Economics, Society and Technology, Brisbane, Queensland, Australia; 3 School of Clinical Sciences, Queensland University of Technology, Brisbane, Queensland, Australia; 4 Center for Research in Economics, Management and the Arts, Zürich, Switzerland; 5 Crawford School of Public Policy, Australian National University, Canberra, Australian Capital Territory, Australia

## Abstract

**Importance:**

Misuse and overselling of over-the-counter pharmaceuticals poses a major burden on both private and public health expenditures.

**Objective:**

To seek evidence on whether over-the-counter medication dispensing behavior complies or conflicts with the protocols indicated in practice standards and guidelines of a national professional pharmacy organization.

**Design, Setting, and Participants:**

This quality improvement study was undertaken in 205 pharmacies in the wider Brisbane, Australia, area. Two standardized patient (SP) scenarios were developed to evaluate noncompliant behavior. Data collection for scenario 1 was conducted between November 23 and December 9, 2016. Data collection for scenario 2 was conducted between September 1 and 28, 2017. A 2-sample test of proportions and a probit regression model were used to evaluate the likelihood of noncompliant treatments and overtreatments in each case scenario. Statistical analysis was performed from January 30 to June 21, 2018, and revised in May 2019.

**Main Outcomes and Measures:**

Outcomes were the observed likelihood of noncompliant treatments and overtreatments. Noncompliance is defined as treatments not complying with practice standards and guidelines set by the professional pharmacy society. Noncompliant treatments include undertreatment (patient did not receive necessary treatment) and overtreatments (patient was supplied with more than sufficient treatments) in both scenarios.

**Results:**

In scenario 1, 9 trained female SPs visited 89 pharmacies to request emergency hormonal contraception from pharmacy staff. In 45 cases, SPs reported having unprotected intercourse within the last 24 hours (case 1A), and in 44 cases, SPs reported having unprotected intercourse more than 72 hours ago (case 1B), which is past the efficacy threshold of over-the-counter emergency hormonal contraception. In scenario 2, 11 SPs (5 male and 6 female) visited 150 pharmacies (154 visits in total) to request treatment for family members or a partner with symptoms indicating bacterial conjunctivitis (case 2A; n = 73) or viral conjunctivitis (case 2B; n = 81). In scenario 1—dispensing emergency hormonal contraception when physician referral is recommended—21 of 44 pharmacists (47.7%) in case 1B violated the recommendation by selling the over-the-counter medication. With the inclusion of both no physician referral and emergency hormonal contraception sold, this rate increased to 79.5% (35 of 44 pharmacists). In scenario 2—1 case each of bacterial and viral conjunctivitis—overtreatment occurred in 55 of 154 cases (35.7%). In both scenarios, 140 of 243 pharmacies (57.6%) followed dispensing behavior compliant with the protocol, while 76 of 243 pharmacies (31.3%) involved some form of overtreatment or overselling of medication. Some evidence of an association between sex of SP and pharmacist was also found.

**Conclusions and Relevance:**

Although the market for dispensing over-the-counter medication in Australia is regulated, relatively high rates of overtreatment and some cases of undertreatment were observed in this study. Given the unintended adverse effects, including overuse of antibiotics and corticosteroids, these observations suggest the advisability of regulatory intervention ensuring compliance with professional protocols.

## Key Points

**Question:**

Are therapeutic guidelines effective mechanisms for ensuring compliance in the provision of over-the-counter pharmaceuticals for symptom-based requests and product-based requests in Australian pharmacies?

**Findings:**

In this quality improvement study using standardized patients in scenarios of requests for emergency hormonal contraception and medication for conjunctivitis, 57.6% of pharmacies followed dispensing behavior compliant with the protocol, while 31.3% involved some form of overtreatment or overselling of medication. There was also evidence of an interaction between sex of the standardized patient and pharmacist.

**Meaning:**

Given the unintended adverse effects of overtreatment, this study suggests the advisability of regulatory intervention (and further behavioral research) to ensure compliance with professional protocols.

## Teaser

This quality improvement study examines whether over-the-counter medication dispensing behavior in Australian pharmacies complies or conflicts with the protocols indicated in practice standards and guidelines of a national professional pharmacy organization.

## Introduction

Having identified both the overuse and the underuse of drugs as pressing dilemmas in general medical expenditures, medical professionals see the need for wide-ranging solutions to be “a moral imperative and a political duty.”^1^^(p191)^ In fact, the frequency with which these problems occur across different markets, countries, and health sectors implies a systematic failure in health care delivery,^1,2,3^ one whose implications have significant effects for public health policy across the globe. A defining feature of modern health care policy as both governments and individuals face mounting medical expenses is thus the push for more autonomy in self-treatment and management. Consequently, alternative health care professionals, such as highly trained pharmacists, are commonly used to ease pressure on frontline services.^4,5^ In fact, community pharmacists, being so highly accessible to the general public, are becoming increasingly more important to frontline health service delivery.^6,7^ This shift is driven not only by increased public reliance on self-medication through over-the-counter products^6,8,9^ and the rapid growth of pharmaceutical expenditures as a share of total medical outlay,^10^ but also by mounting financial pressure on other health services and the underuse of highly trained pharmacists.^4^

One core issue in addressing the overtreatment and undertreatment problem is that health care delivery has considerable information asymmetries between expert clinicians who know more about the type of goods or services the patient needs and the patients or customers who know less about the extent or quality of goods available.^11,12,13,14^ Because the discovery costs of overtreatment or undertreatment are high, such asymmetry can result in overprovision and underprovision of treatment. The pharmacist environment is unique in health care services in that it offers a single point of advice and medication provision. Patients describe symptoms and identify a need, which is the basis for pharmacists to then provide a diagnosis (within certain guidelines) and determine the appropriate intervention (including physician referral if warranted). The pharmacist as expert therefore provides information that informs and influences the customer’s purchasing decision. Yet, according to a recent overview of the related literature, despite improved understanding of the frequency and causes of overuse, the research is poorly coordinated and has yet to answer several core questions pertinent to improving patient care.^15^

We therefore conducted a quality improvement study using standardized patients (SPs) and applied 2 different scenarios to assess pharmacy staff compliance with the professional protocols as a clinical benchmark. Research assistants trained as SPs (customers looking for treatments based on the scenario) made more than 230 visits to 205 different pharmacies to collect interaction data. Our research setting was Brisbane, Australia, which has seen major growth in medical costs to both governments and individuals during the past 20 years, with subsidized pharmaceutical expenditures in 2013-2014 exceeding A$10.1 billion (US $7.1 billion), second only to general medical services. During the same period, spending on prescribed medications and over-the-counter medications was A$9.7 billion (US $6.8 billion), 93% of it paid by individuals.^16^ In 2014, the market for all prescription and over-the-counter medicines was worth more than A$19.8 billion (US $13.9 billion), which equated to 1.4% of Australia’s gross domestic product. Hence, although Australian pharmacies (like other health services) are heavily regulated by Pharmaceutical Society of Australia standards for the provision of medicines and appropriate advice,^17^ the asymmetric information structure could still encourage undertreatment or overtreatment.

The aim of this study was to examine both diagnostic and compliance behavior of pharmacists in the treatment and provision of medication for 2 common scenarios (a request for emergency hormonal contraception [EHC] and treatment for conjunctivitis) using SPs in the actual field. However, pharmacists are not required to comply with said guidelines as if they are a fixed protocol; rather, they are to be used as a set of clinical practice guidelines to assist in clinical decision-making.

## Methods

### Study Design and Setting

For this quality improvement study, conducted in Brisbane, Australia, between November 23, 2016, and September 28, 2017, we drew on best-practice material from the *Therapeutic Guidelines and Australian Medicines Handbook* to construct 2 scenarios representing ideal case management.^18,19^ These guidelines are developed by expert panels in each subject area together with associated member organizations of NPS MedicineWise (programs funded by the Australian Federal Department of Health), the Australian Medical Association, the Royal Australian College of General Practitioners, the Pharmaceutical Society of Australia, and the Pharmacy Guild of Australia. Supporting evidence for these case management ideals are provided in the Pharmaceutical Society of Australia’s *Guidance for Provision of a Pharmacist Only Medicine* and *Professional Practice Standards*.^20^ We outline the case descriptions, SP scenarios, and expected or ideal guideline-based case management in eTable 1 and eTable 2 in the Supplement. The SP scenarios also considered pharmacist sex, type of pharmacy location (strip mall, shopping center, stand-alone, or medical center), and total number of questions asked. For scenario 2, we additionally note whether or not the pharmacist asked key diagnostic questions to identify the proper treatment.

Ethical approval for the study was granted by the Queensland University of Technology University Human Research Ethics Committee on October 4, 2016. The Queensland University of Technology Ethics Committee granted approval for a waiver of consent of study participants prior to data collection because participants were not informed prior to observation so as not to bias (pharmacist) behavior with the knowledge that they were being observed. This method of research is commonly used in the pharmacy profession for quality assurance purposes. In addition, the ethics review approval provisioned that both relevant regulatory bodies (Pharmacy Guild and Pharmaceutical Society of Australia) were sent the study (ex post completion) for dissemination in their regular member bulletins.

### Standardized Patients

A total of 34 undergraduate business students between the ages of 19 and 29 years were recruited to act as SPs, all of whom received appropriate training from the clinical scientists in our research team (G.K. and E.T.L.L.) before field data collection began. Pharmacy students were not used because the university’s Clinical Sciences Department has a close relationship with community pharmacies, which provide the students with work placements at the end of their first year. These students recruited as SPs were given a uniform script for their allocated scenario and were required to fill out a survey immediately after leaving the pharmacy to avoid any recall biases (see the eAppendix in the Supplement for the survey questionnaire and detailed protocols).

### Pharmacy Selection, SP Visits, and Study Size

Because the research university is centrally located in the central business district, with no onsite accommodation on the central business district campus, the students’ residential locations were almost random, which allowed their allocation to pharmacies conditional on area of residence (eFigure in the Supplement). Data collection for scenario 1 (EHC) was conducted from November 23 to December 9, 2016. A total of 9 female SPs, assigned to minimize the confounding effects of the medication being sex specific, made 1 visit each to 89 pharmacies in the wider Brisbane area (eFigure, A, in the Supplement). Scenario 2 (conjunctivitis) was carried out from September 1 to 28, 2017. A total of 11 SPs (5 male and 6 female) visited 154 pharmacies (eFigure, B, in the Supplement), 4 of which were visited twice (once for each treatment). Across the entire phase, 38 pharmacies received 2 visits.

### SP Scenarios

#### Scenario 1: EHC

At the time of this study, the most widely used and cost-effective form of EHC was a 1.5-mg tablet of levonorgestrel.^1^ Although levonorgestrel was originally categorized by the Therapeutic Goods Authority as an S4 (prescription-only) drug, it was downgraded in January 2004 to an S3 (pharmacist-only) medicine to reduce barriers to supply.^21^ The efficacy of this synthetic drug, which replicates the natural hormone progesterone and works by interrupting ovulation,^20^ depends on the time interval between intercourse and ingestion, with accepted rates of efficacy based on a multicenter double-blind randomized clinical trial by the World Health Organization.^22^ Levonorgestrel has a short half-life (eTable 3 in the Supplement), and there is little to no evidence of its effectiveness if it is taken more than 72 hours after unprotected intercourse.^23^ Hence, the clinical and therapeutic guidelines for pharmaceutical dispensation of EHC, which are based on observed efficacy levels at relevant time intervals (eTable 3 in the Supplement), restrict levonorgestrel to 1.5 mg administered within 72 hours after unprotected intercourse.^18,19^ Provision of this drug after 72 hours is strictly off label (use outside its own stated consumer medicine information) and unapproved by government regulators because of reduced efficacy and because postcoital contraception with higher efficacy rates after 72 hours are available, albeit only as administered or prescribed by a physician.^20^

The challenge presented by this scenario is that the diagnosis is uncertain because of a complete lack of physical symptoms and no means of confirming whether an egg has been fertilized and implanted within 3 to 4 days. It is impossible to know whether the patient would have become pregnant if she had not sought treatment, and because the drug is designed to prevent implantation by interrupting ovulation, the intervention must occur before the pregnancy actually begins (ie, preoutcome).

We applied the scenario to 2 cases. In case 1A, EHC was requested when unprotected intercourse happened within less than 24 hours, referred to in the scenario script as “last night.” In case 1B, EHC was requested when unprotected intercourse happened more than 72 hours ago, referred to as “probably 3 days ago last night.” This latter case creates a dilemma in that following the *Australian Medicines Handbook* guideline of referral to a general practitioner without EHC provision results in no sale, and thus no revenue, for the pharmacy. In Table 1, we provide a systematic breakdown of compliance between high-quality (fully compliant) and low-quality (inefficient) treatment subject to undertreatment and overtreatment.

**Table 1.** Scenario 1: EHC Treatment Compliance for Direct Product Request

Compliance Outcome	Case 1A: Direct Product Request^a^		Case 1B: Direct Product Request^b^	
	Referral to Physician	EHC Provided	Referral to Physician	EHC Provided
High-quality care				
Compliant	No	Yes	Yes	No
Low-quality care				
Undertreatment 1	No	No	No	No
Undertreatment 2	NA	NA	No	Yes
Overtreatment 1	Yes	Yes	Yes	Yes
Overtreatment 2	Yes	No	NA	NA

Abbreviations: EHC, emergency hormonal contraceptive; NA, not applicable.

^a^ Unprotected intercourse within 24 hours.

^b^ Unprotected intercourse more than 72 hours ago.

#### Scenario 2: Infective Conjunctivitis

Conjunctivitis, an inflammation or infection of the mucous membrane covering the anterior sclera and inside eyelid, has 2 infectious forms, viral and bacterial, as well as a noninfectious form induced by allergens. Acute conjunctivitis is a relatively common ailment, with an estimated incidence reported in primary care settings of approximately 1.5% to 2% in the developed world.^24^ Although viral and bacterial conjunctivitis present with differentiating characteristics (eTable 4 in the Supplement), some studies suggest that pathogenic ambiguity can lead to misdiagnoses in as high as half of all cases,^25^ meaning that many patients receive inappropriate treatment. However, the unnecessary provision of antibiotics to individuals with the viral rather than the bacterial form of conjunctivitis adds to the development of antimicrobial resistance from overprescription of antibiotics. In reality, most conjunctivitis cases are self-limiting within 1 to 2 weeks of presentation, with minimal risk of long-term vision loss,^19,26^ a spontaneous remission rate that has led to increasing encouragement of a “delayed prescription” or “delayed antibiotic” approach.^25^

Because in our scenario the visual signs and symptoms for viral and bacterial conjunctivitis precluded the SPs from requesting treatment for themselves, the SPs requested treatment for a family member or partner.^27^ For case 2A, when symptoms indicate bacterial conjunctivitis, the ideal management specified in the guidelines is prescription of a topical ocular antibiotic or, for mild cases, antiseptic eye drops (Table 2). Ocular lubricants or saline solutions will not treat the infection, although they can relieve some discomfort. Case 2B describes the presentation of viral conjunctivitis for which antiviral drugs are not recommended; rather, treatment is symptomatic and includes saline solution or an ocular lubricant. Diagnostic ambiguity is present because of conspecific symptom overlap between viral conjunctivitis and the most common type of noninfectious conjunctivitis, allergic conjunctivitis, whose incidence is very high. In the empirical analysis, we controlled for the contradistinctive symptoms of itchy or burning eyes and/or a history of allergies by asking the SP (posing as a family member or partner) the following diagnostic questions: “Do they have any allergies?” (with an answer of “No”) and “Do they have any other symptoms?” (with an answer of “Yes, they had a cold about a week ago”) (eTable 4 in the Supplement).

**Table 2.** Scenario 2: Conjunctivitis Treatment Compliance for SBR

Compliance Outcome	Case 2A: SBR (Bacterial Conjunctivitis)	Case 2B: SBR (Viral Conjunctivitis)
High-quality care		
Compliant	Antibiotic or antibacterial eye drops or ointment	Ocular lubricants or saline solution
Low-quality care		
Undertreatment 1	No treatment^a^	No treatment^a^
Undertreatment 2	Ocular lubricant or saline solution	NA
Overtreatment 1	≥2 Treatments	Antibiotic or antibacterial eye drops or ointment
Overtreatment 2	NA	Antihistamine and/or decongestant
Overtreatment 3	NA	≥2 Treatments

Abbreviations: NA, not applicable; SBR, symptom-based request.

^a^ Excluding refusal when pharmacy could not assess patient (third-party refusal).

### Outcome Measures

The 2 outcome variables were pharmacy staff providing inefficient treatments (as opposed to full treatment compliance) and overtreatments based on the case management criteria for each scenario and case defined in Table 1 and Table 2. We included the full staff and SP sex interaction in product terms (male/female staff × male/female SP) to understand whether staff sex is associated with SP sex.

### Statistical Analysis

Statistical analysis was performed from January 30 to June 21, 2018, and revised in May 2019. Data were analyzed separately for the EHC scenario and for the infective conjunctivitis scenario. A 2-sample test of proportions was used to evaluate the frequency of outcomes between cases, and multivariate regression was used to assess the contributing factors. Case 1A (unprotected intercourse within the last 24 hours) in scenario 1 was not included in the regression analyses because full compliance was observed for each observation within case 1A. Probit regression models were used to assess factors associated with noncompliance and overtreatments including pharmacy-specific characteristics (store location, banner group, and price match policy), pharmacist-specific variables (sex and the number of questions asked), and SP-specific variables (sex and time of visit). The term *banner group* is specific to the Australian market and refers to franchisee partnerships between upstream wholesalers and operating pharmacists. For scenario 2 (conjunctivitis), we included the full staff and SP sex interaction terms to explore sex effects. For the multivariate analyses, we estimated the quantitative associations of each variable by reporting the mean marginal effect (ie, the mean change in probability of observing the outcome when the variable increases by 1 unit [continuous variable] or compared with the reference group [categorical variable] of a probit model). Standard errors of the mean marginal effect were clustered on retail group levels. The coefficient estimates of the models are presented in eTable 5 in the Supplement. All statistical analyses were conducted in Stata, version 15.1 SE (StataCorp). Statistical significance is reported at 10%, 5%, 1%, and 0.1% levels with 2-sided tests. We consider the coefficient to be statistically significant if *P* < .01.

## Results

### Characteristics of the Samples

Of the 243 visits, 168 (69.1%) were to pharmacies belonged to a banner group and 99 (40.7%) were to pharmacies that had a price match policy. The pharmacies visited were located in strip malls (107 visits [44.0%]), shopping centers (90 visits [37.0%]), or medical centers (26 visits [10.7%]) or were stand-alone shops (20 visits [8.2%]). Of the 243 pharmacy staff members who interacted with the SPs (70.8%) were male and 71 (29.2%) were female. The median of number of diagnostic questions asked was 6 (interquartile range, 3-8 questions per visit). Of the 243 visits, 165 (67.9%) were in the afternoon (between 12 and 4 pm), 43 (17.7%) were in the morning (before 12 pm), and 35 (14.4%) were at night (after 4 pm). A total of 96 visits (39.5%) visits were between Friday and Sunday and 147 (60.5%) were between Monday and Thursday. In both scenarios, 140 of 243 pharmacies (57.6%) followed dispensing behavior compliant with the protocol, while 76 of 243 pharmacies (31.3%) involved some form of overtreatment or overselling of medication.

### Scenario 1: EHC

**Figure 1.**
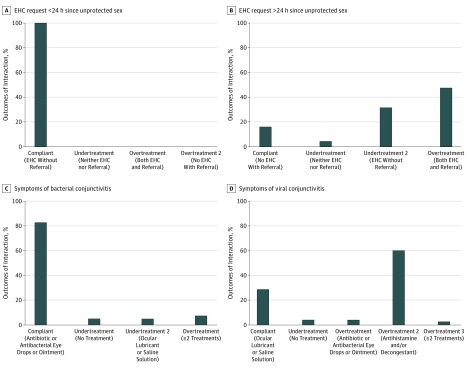
Distribution of Observed Treatment Outcomes by Scenario A, Scenario 1: emergency hormonal contraceptive (EHC) request less than 24 hours since unprotected sex (n = 45). B, Request for EHC more than 24 hours since unprotected sex (n = 44). C, Scenario 2: conjunctivitis symptoms indicative of bacterial conjunctivitis (n = 81). D, Conjunctivitis symptoms indicative of viral conjunctivitis (n = 73). Compliant, overtreatment, and undertreatment conditions are as defined in Table 1.

For scenario 1, although the pharmacy staff followed the recommended treatment guidelines in all case 1A observations (n = 45), there was far more variation in case 1B (n = 44), in which the experimental parameter of more than 72 hours elapsed since intercourse indicated physician referral as the primary choice for ideal management. Moreover, although 28 of 44 case 1B interactions (63.6%) included referrals, only 7 (15.9%) of these referrals provided high-quality care (physician referral and no EHC), while 21 (47.7%) resulted in overtreatment (physician referral and off-script provision of EHC). With the inclusion of both no physician referral and EHC sold, 35 of 44 pharmacists (79.5%) did not follow the recommended treatment guidelines. In 3 nonefficient (either undertreated or overtreated) case 1B observations, the SP documented that the pharmacy staff emphasized the low efficacy of the drug but supplied it anyway. In most (14 of 16 [87.5%]) observed undertreatments (no physician referral), the pharmacy expert provided the medicine off-script (Figure 1).

For case 1A, 38 of 44 interactions (84.4%) included a discussion of treatment options, 23 (51.1%) included follow-up advice, and 9 (20.0%) included advice on lifestyle modifications. For case 1B, conditional on sale of EHC, only 24 of 35 interactions (68.6%) included a discussion of treatment options, and although 21 interactions (60.0%) included advice on follow-up, only 4 interactions (11.4%) included advice on lifestyle modifications.

### Scenario 2: Infective Conjunctivitis

For this clinical presentation, although antibiotics and antibacterials are interchangeable as appropriate treatments in case 2A (bacterial conjunctivitis; n = 81), antihistamines and/or decongestants are inappropriate in case 2B (viral conjunctivitis; n = 73). We can thus determine the type of conjunctivitis diagnosed based on the clinical applications of the products supplied. Overtreatment occurred in 55 of 154 cases (35.7%). For scenario 2, the SPs recorded 154 observations, 7 of them resulting in no sale (Figure 1). In 10 interactions, the pharmacy staff recommended more than 1 treatment; for example, 1 sale in case 2A included both the eye drops and eye ointment of the same antibiotic product. Moreover, even though 67 of the interactions (82.7%) in case 2A were compliant with therapeutic guidelines, this rate decreased to 28.8% (n = 21) in case 2B. The potential diagnostic complexities for pharmacists are underscored by the overtreatment rate, which was statistically significant between cases 2A (6 of 81 [7.4%]) and 2B (49 of 73 [67.1%]) (based on a 2-sample, 2-tailed test of proportions; *P* < .001). Likewise, the differences between the 2 scenarios clearly indicated that the condition’s differential aspects are embedded in the interaction.

For case 2B, the pharmacist must differentiate between the highly similar presentations of viral conjunctivitis and allergic conjunctivitis, making 2 questions integral to the diagnosis: “Do they have any allergies?” and “Do they have any other medical conditions?” The answers supplied by the SPs (no history of allergies and the patient having had influenza “about 2 weeks ago”) point to a high probability of viral conjunctivitis. However, in the cases of compliant behavior recorded (n = 31) for case 2B, only 2 interactions included both these questions, and only 4 included 1 of the questions. Assumedly, in this scenario, the similarity of viral conjunctivitis to other topical types, particularly the highly prevalent allergic conjunctivitis, led pharmacists and pharmacist assistants to recommend antihistamines as the primary treatment in 33 of the 70 interactions (47.1%) involving a sale. However, of the 49 interactions that led to overtreatment, 33 (67.3%) included at least 1 of the critical diagnostic questions compared with only 6 (28.6%) among the 21 compliant interactions.

### Sex in Diagnosis and Treatment

**Figure 2.**
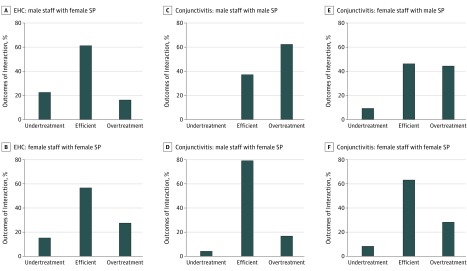
Staff and Standardized Patient Sex Interactions by Scenario A, Emergency hormonal contraceptive (EHC) case for male staff with female standardized patient (SP). B, Emergency hormonal contraceptive case for female staff with female SP. C, Conjunctivitis case for male staff with male SP. D, Conjunctivitis case for male staff with female SP. E, Conjunctivitis case for female staff with male SP. F, Conjunctivitis case for female staff with female SP.

From a broader social perspective, it is also important to note the role in patient treatment of behavioral considerations associated with sex (Figure 2), particularly in the case of over-the-counter EHC (scenario 1). However, although the intimate nature of a request for EHC from a female buyer might be expected to increase the likelihood of a male pharmacist referring the patient to a female colleague, we observed no such behavior in our sample, even though this scenario involved the highest percentage of primary contact with male pharmacy staff (34.8% [31 of 89] compared with 26.0% [40 of 154] for scenario 2). Although overtreatment occurred more often when SPs interacted with female staff (5 of 31 [16.1%] for interaction with male staff compared with 16 of 58 [27.6%] for interaction with female staff in both cases in scenario 1 and 5 of 15 [33.3%] for interaction with male staff compared with 16 of 29 [55.2%] for interaction with female staff in case 1B alone), there was no statistically significant difference in the proportion of overtreatment outcomes in scenario 1 (2-sample, 2-tailed test of proportions; *P* = .23 and *P* = .17 when limiting to only case 1B). The interactions in scenario 2, in contrast, with their near-random assignment of male and female SPs, bring to light more sex variations. When the SP was male, for instance, the results showed a significant association between sex and overtreatment in both the pooled (male SP interaction, 34 of 70 [48.6%]; and female SP interaction, 21 of 84 [25.0%]; *P* = .002) and split samples of female (10 of 16 [62.5%] for female staff and male SP interaction; and 4 of 24 [16.7%] for female staff and female SP interaction; *P* = .07) and male staff (34 of 70 [48.6%] for male staff and male SP interaction; and 21 of 84 [25.0%] for male staff and female SP interaction; *P* = .003). Likewise, the share of overtreatment for male SPs in both same-sex and opposite-sex interactions was statistically significantly higher than that for female SPs.

### Multivariate Results

Table 3 presents the multivariate analyses. Pharmacies belonging to a banner group were less likely to provide noncompliant treatment or overtreatment in all scenarios (ranging from 14% to 35.3%), while stores with a price match policy were more likely to provide noncompliant treatment or overtreatment, particularly for scenario 1 (ie, 32.6% and 43.4%). Store location was associated with a small degree of behavioral compliance; stores at a shopping center were more likely to be compliant in scenario 2 than were stores at strip mall locations. Interacting only with a pharmacist (as opposed to a pharmacy assistant or when being referred from an assistant to pharmacist) reduced the likelihood of receiving noncompliant treatment in scenario 2. Similar to these findings, the female SPs in case 1B were 23.3% more likely to receive overtreatment from a female staff member compared with a male staff member (*P* = .002). However, we did not find a statistically significant sex interaction effect in scenario 2. Total number of questions asked was positively associated with the probability of receiving overtreatment in both scenarios (and overall noncompliant treatment in case 1B). Compared with a case in which no diagnostic questions were asked (specifications 5 and 6), asking only 1 key diagnostic question had a statistically significant negative association with compliance, which nonetheless signals an increased chance of noncompliant diagnosis and treatment for the patient. Visits during the evening (after 4 pm) resulted in a decreased likelihood of noncompliant treatment (27.8%) and overtreatment (30.7%) for EHC (when requested >72 hours after intercourse) compared with afternoon visits (between 12 and 4 pm). Such an association was weaker and not statistically significant for scenario 2. Visits for EHC on a weekend (as opposed to Monday to Thursday) also led to a statistically higher rate (14.1%) of noncompliant treatment (ie, no referral to a physician and/or EHC provision) but not overtreatment (physician referral and EHC provision). Day of the week did not have a statistically significant association with quality of treatment received in scenario 2. Finally, higher diagnosis ambiguity (case 2B in scenario 2) had a higher rate of noncompliant treatment and overtreatment.

**Table 3.** Multivariate Analysis (Probit Regression) of Factors Associated With Noncompliance

Scenario	Mean Marginal Effect (SE)					
	Emergency Hormonal Contraceptive Case 1B		Infective Conjunctivitis			
	Noncompliant	Overtreatment	Noncompliant^a^	Overtreatment^a^	Noncompliant^b^	Overtreatment^b^
Banner group	−0.32 (0.08)^c^	−0.35 (0.06)^d^	−0.23 (0.03)^d^	−0.14 (0.04)^e^	−0.23 (0.03)^d^	−0.14 (0.04)^e^
Price match policy	0.33 (0.12)^c^	0.43 (0.05)^d^	0.16 (0.04)^d^	0.09 (0.04)^c^	0.16 (0.04)^d^	0.07 (0.03)^c^
Location						
Strip mall	[Reference]	[Reference]	[Reference]	[Reference]	[Reference]	[Reference]
Shopping center	−0.06 (0.13)	−0.14 (0.08)	−0.14 (0.06)^c^	−0.04 (0.08)	−0.14 (0.06)^c^	−0.05 (0.09)
Stand-alone	Omitted^f^	−0.35 (0.14)^g^	−0.22 (0.13)^g^	−0.08 (0.10)	−0.23 (0.12)^c^	−0.10 (0.09)
Medical center	−0.10 (0.04)^g^	0.01 (0.17)	−0.06 (0.13)	0.03 (0.11)	−0.08 (0.12)	0.01 (0.10)
Interaction with pharmacist only	−0.02 (0.05)	−0.16 (0.06)^g^	−0.07 (0.02)^e^	−0.05 (0.03)^g^	−0.08 (0.03)^e^	−0.03 (0.02)
Interaction						
Male staff and male SP	[Reference]	[Reference]	[Reference]	[Reference]	[Reference]	[Reference]
Female staff and female SP	0.10 (0.16)	0.23 (0.07)^e^	0.09 (0.22)	−0.06 (0.18)	0.09 (0.21)	−0.03 (0.18)
Male staff and female SP	NA	NA	0.05 (0.10)	−0.05 (0.08)	0.07 (0.11)	0.02 (0.07)
Female staff and male SP	NA	NA	0.14 (0.09)	−0.00 (0.06)	0.12 (0.09)	0.01 (0.06)
No. of questions asked	0.03 (0.01)^e^	0.10 (0.02)^e^	−0.00 (0.02)	0.02 (0.01)^c^	NA	NA
No. of diagnostic questions asked						
None	[Reference]	[Reference]	[Reference]	[Reference]	[Reference]	[Reference]
1	NA	NA	NA	NA	0.15 (0.07)^c^	0.15 (0.06)^e^
Both diagnostic questions	NA	NA	NA	NA	0.03 (0.09)	0.03 (0.12)
Time of day						
Morning (before 12 pm)	−0.01 (0.16)	−0.04 (0.07)	0.02 (0.13)	−0.17 (0.08)^c^	0.03 (0.12)	−0.15 (0.08)^g^
Afternoon (12-4 pm)	[Reference]	[Reference]	[Reference]	[Reference]	[Reference]	[Reference]
Evening (after 4 pm)	−0.28 (0.14)^c^	−0.31 (0.06)^e^	−0.06 (0.11)	−0.04 (0.11)	−0.08 (0.11)	−0.04 (0.11)
Day of week						
Monday to Thursday	[Reference]	[Reference]	[Reference]	[Reference]	[Reference]	[Reference]
Friday to Sunday	0.14 (0.05)^c^	0.15 (0.11)	0.08 (0.09)	0.08 (0.05)	0.09 (0.10)	0.06 (0.04)
Scenario 2, case 2B (viral conjunctivitis)	NA	NA	0.55 (0.10)^d^	0.60 (0.10)^d^	0.54 (0.09)^d^	0.55 (0.10)^d^
Observations, No.	41	44	154	154	154	154
Clusters, No.	7	7	7	7	7	7
Pseudo-*R*^2^	0.352	0.573	0.323	0.381	0.339	0.387

Abbreviations: NA, not applicable; SP, standardized patient.

^a^ Controlling for total number of questions asked.

^b^ Controlling for number of diagnostic questions asked.

^c^ *P* < .05.

^d^ *P* < .001.

^e^ *P* < .01.

^f^ Omitted because noncompliant behavior was observed in all 3 stand-alone pharmacies. Robust standard errors of average marginal effects were clustered on retail group levels.

^g^ *P* < .10.

## Discussion

As with any other expert assistance, the unique and often highly pressured frontline health services provided by pharmacists can be influenced by information transfers in social interactions and by the psychology and emotional state of others. The 2 scenarios in this study illustrate this influence through very different yet challenging diagnosis and provision problems in the pharmacy setting. In the EHC case 1B scenario, the provision problem for pharmacy staff, although centered on the customer’s physical health, tends to also carry an emotional cost related to the nature of the condition described. The documentation provided by one SP in case 1B provides qualitative insight into such emotional concerns and how they color motivations: “The pharmacist was initially not going to supply it [EHC] because of the time elapsed; however, after I said I didn’t mind, whatever they recommended, they then sold it to me because it was a Friday and she was worried I would not be able to get to the GP [general practitioner].” Nonetheless, in our sample, a sale being made in this case is not likely to be conditional on the presentation occurring on or near the weekend (ie, Friday) when general practitioners are less available and time-sensitive considerations increase. Rather, over-the-counter EHC provision to women by pharmacy experts comes not only with an evaluation dilemma tied to duration since intercourse but, to a lesser extent, with some level of empathy and emotional support in its provision to the customer. This emotional influence may create complications in effective provision. Although there can be no doubt that pharmaceutical provision encompasses a level of empathy and understanding for patients and their medical issues or health, this study provides a very unique SP scenario of reproductive health unlike any previous recent SP studies, to our knowledge.^27,28^

In our infective conjunctivitis scenario, pharmacists faced a different issue in efficient provision, that of effective diagnosis. Because the focal point of any symptom-based request is an expert’s ability to both listen and ask the right questions, the scripts provided for cases A and B in this scenario were carefully crafted to facilitate the correct diagnosis of the condition described. Hence, the high prevalence of inefficient provision in our sample highlights the information asymmetries in the communication process between the expert clinician and the patient. Such findings align with previous research in that information asymmetry and any subsequent overtreatment can often be attributed to both complexity in diagnosis or simply failure to ask relevant questions to facilitate a successful diagnosis.^27,28^

### Limitations

There are 4 main limitations to this study. First, the scenarios selected may not be generalized to the provision of other types of over-the-counter medication. Standardized patients were university undergraduate students from Queensland University of Technology recruited for this study, so inferences drawn may not represent the interactions with other members of the population (eg, different age cohorts). Second, the frequency of noncompliant treatment may be influenced by seasonality or demand (eg, influenza season), which is not well accounted for in our study because data were collected during a short period of time (a few weeks). In particular, research has shown that demand for EHC is substantially higher during weekends, the summer holiday, and major festivals, which is likely the result of increased (unsafe) sexual activities.^29^ Although all data collection for scenario 1 was conducted within the Queensland school term with no major festival, we observed an association between the weekend effect and noncompliant behavior. Third, the present study did not capture comparative data on patient-physician interaction and compliance behavior. It is unclear if, for example, overuse of EHC by pharmacists is simply a supply response to market forces imposed by physician behavior. Fourth, it must be noted that there is a gamut of external information (eg, aesthetic, emotive, or contextual factors) involved in the pharmacist-patient interaction that simply cannot all be captured entirely for the purposes of such quality improvement studies.

## Conclusions

As the most readily available frontline health service to the public, pharmacies have begun playing a pivotal role in consumer health needs, prompting the Pharmaceutical Society of Australia in 2016 to argue that these highly skilled but underused professionals should play a role in reducing the burden of increasing health costs across the sector.^30^ Understanding the interplay of the myriad factors relevant in diagnosis and treatment provision in this setting is thus critical to ensuring effective and efficient outcomes for all parties as the pharmacy market moves forward. However, this study’s reliance on markup value restricted our ability to analyze firm-size costs. Future studies may thus seek access to more accurate data on this factor to provide a more robust analysis.

## Notes

Supplement. **eTable 1.** Standardized Patient Case Descriptions—Emergency Hormonal Contraceptive **eTable 2.** Standardized Patient Case Descriptions—Infective Conjunctivitis **eTable 3.** Efficacy—Emergency Hormonal Contraceptive (EHC) **eTable 4.** Symptoms and Indicators of Bacterial vs Viral Conjunctivitis **eTable 5.** Multivariate Analysis (Probit Regression) of Factors Impacting Noncompliance **eFigure.** Distribution of Pharmacies Visited **eAppendix.** Research Assistant Checklist
