# Supplementary material for: Pharmacist Compliance With Therapeutic Guidelines on Diagnosis and Treatment Provision
Source: JAMA Netw Open. 2019 Jul 17;2(7):e197168. doi: 10.1001/jamanetworkopen.2019.7168 (PMC6647553; doi:10.1001/jamanetworkopen.2019.7168)
Supplement: Supplement. — eTable 1. Standardized Patient Case Descriptions—Emergency Hormonal Contraceptive eTable 2. Standardized Patient Case Descriptions—Infective Conjunctivitis eTable 3. Efficacy—Emergency Hormonal Contraceptive (EHC) eTable 4. Symptoms and Indicators of Bacterial vs Viral Conjunctivitis eTable 5. Multivariate Analysis (Probit Regression) of Factors Impacting Noncompliance eFigure. Distribution of Pharmacies Visited eAppendix. Research Assistant Checklist [file jamanetwopen-2-e197168-s001.pdf]

Supplementary Online Content

Smith H, Whyte S, Chan HF, et al. Pharmacist compliance with therapeutic guidelines on diagnosis and treatment provision. *JAMA Netw Open.* 2019;2(7):e197168. doi:10.1001/jamanetworkopen.2019.7168

eTable 1. Standardized Patient Case Descriptions—Emergency Hormonal Contraceptive

eTable 2. Standardized Patient Case Descriptions—Infective Conjunctivitis

eTable 3. Efficacy—Emergency Hormonal Contraceptive (EHC)

eTable 4. Symptoms and Indicators of Bacterial vs Viral Conjunctivitis

eTable 5. Multivariate Analysis (Probit Regression) of Factors Impacting Noncompliance

eFigure. Distribution of Pharmacies Visited

eAppendix. Research Assistant Checklist

This supplementary material has been provided by the authors to give readers additional information about their work.

© 2019 Smith H et al. *JAMA Network Open.*

eTable 1. Standardized Patient Case Descriptions—Emergency Hormonal Contraceptive

	Case Description	Standardised Patient Presentation	Expected Case management
Case 1A	Emergency contraception hormone request <24 hours since unprotected sex	SP presents with the opening statement: "Hi, I need the morning after pill." **When did you have sex?** Last night. **When is the last time you had your period?** I think it was about 2 weeks ago. **Do you have any medical conditions?** No. **Are you on any other medication?** No. **Do you have any allergies?** Not that I know of.<br><br>**If asked:** Have not taken a pregnancy test recently. Not pregnant or breastfeeding. No burning or pain when passing urine, no abdominal pain, no pain during or after sex, no unusual vaginal discharge, no irregular bleeding. Just the one incident of unprotected sex; no other unprotected sex any other time. I don't think I'm at risk of STIs, but will see my doctor to get a test done.	Provision of EHC
Case 1B	Emergency contraception hormone request >72 hours since unprotected sex	SP presents with the opening statement: "Hi, I need the morning after pill." **When did you have sex?** I guess it would be three nights ago last night. **When is the last time you had your period?** I think it was about 2 weeks ago. **Do you have any medical conditions?** No. **Are you on any other medication?** No. **Do you have any allergies?** Not that I know of.<br><br>**If asked:** Have not taken a pregnancy test recently. Not pregnant or breastfeeding. No burning or pain when passing urine, no abdominal pain, no pain during or after sex, no unusual vaginal discharge, no irregular bleeding. Just the one incident of unprotected sex, no other unprotected sex any other time. I don't think I'm at risk of STIs, but will see my doctor to get a test done.	No provision of EHC & referral to a GP.

© 2019 Smith H et al. *JAMA Network Open.*

eTable 2. Standardized Patient Case Descriptions—Infective Conjunctivitis

	Case Description	Standardised Patient Presentation	Expected Case management
Case 2A	Symptoms indicative of BACTERIAL conjunctivitis	SP presents with the opening statement: "Hi, my family member/partner has a sore left/right eye that they could not open this morning as there was "gunk" sticking it together".<br><br>**When did this begin?** This morning. **What colour was the discharge?** It was slightly yellow and crust was a browning colour. **Do they wear contact lenses?** No. **Have they had eye surgery/laser treatment recently?** No. **Have they been treated for this before?** No. **Do they have any other symptoms [they may ask about flu or hayfever**]? No. **Do they have any allergies?** No. **Are they on any other medication?** No.<br><br>**If asked:** They do not have a family history of glaucoma. They have not recently travelled overseas. It is not painful. They do not have light sensitivity. It is not itchy. Their vision has not changed.	Antibiotic or Antibacterial eye drops
Case 2B	Symptoms indicative of VIRAL conjunctivitis	SP presents with the opening statement: "Hi, my family member/partner has red sore eyes. Their eyes wouldn't stop watering and were irritated".<br><br>**When did this begin?** This morning. **What colour was the discharge?** Red eyes, but clear discharge. **Do they wear contact lenses?** No. **Have they had eye surgery/laser treatment recently?** No. **Have they been treated for this before?** No. **Do they have any other symptoms [they may ask about flu or hayfever**]? Yes, had a cold about a week ago.<br>**Do they have any allergies?** No. **Are they on any other medication?** No.<br><br>**If asked:** They do not have a family history of glaucoma. They have not recently travelled overseas. It is not painful. They do not have light sensitivity. It is not itchy and they can open their eyes. Their vision has not changed.	Ocular lubricant or Saline solution

© 2019 Smith H et al. *JAMA Network Open.*

eTable 3. Efficacy—Emergency Hormonal Contraceptive (EHC)

Hours after intercourse	Efficacy of drug
0-24	95%
25-48	85%
49-72	58%

© 2019 Smith H et al. *JAMA Network Open.*

eTable 4. Symptoms and Indicators of Bacterial vs Viral Conjunctivitis

**Infective Conjunctivitis**	**Signs or Symptoms**	**Not Indicators**
Bacterial	Purulent discharge, matting of eyelids, redness, swelling, usually originates in one eye, eyelid swelling	Chemosis, itching, photophobia, vision change
Viral	Watery discharge, itchiness, redness, recent or current cold/flu or upper respiratory tract infection, eyelid swelling	Chemosis, itching, vision change

© 2019 Smith H et al. *JAMA Network Open.*

eTable 5. Multivariate Analysis (Probit Regression) of Factors Impacting Noncompliance

	**Regression coefficients (95% CI)**					
**Scenario**	**Emergency Hormonal Contraceptive (EHC Case 1B)**		**Infective Conjunctivitis (EYE)**			
**Outcome Factor**	**Non-compliant**	**Over-treatment**	**Non-compliant**	**Over-treatment**	**Non-compliant**	**Over-treatment**
Banner Group	-2.31 (-4.07,-0.54)[c]	-2.68 (-4.08,-1.27)[a]	-0.92 (-1.20,-0.63)[a]	-0.65 (-1.07,-0.23)[b]	-0.95 (-1.28,-0.62)[a]	-0.65 (-1.11,-0.18)[b]
Price Match Policy	2.44 (0.40,4.49)[c]	2.85 (1.45,4.26)[a]	0.63 (0.27,0.99)[a]	0.38 (0.05,0.70)[c]	0.61 (0.28,0.93)[a]	0.30 (0.03,0.58)[c]
**Location**						
Strip mall	[Reference]	[Reference]	[Reference]	[Reference]	[Reference]	[Reference]
Shopping Centre	-0.37 (-1.93,1.19)	-1.00 (-2.25,0.26)	-0.53 (-0.98,-0.08)[c]	-0.17 (-0.92,0.58)	-0.54 (-1.03,-0.05)[c]	-0.22 (-0.98,0.53)
Stand Alone	Omitted[e]	-2.58 (-5.62,0.45)[d]	-0.85 (-1.84,0.14)[d]	-0.38 (-1.21,0.46)	-0.93 (-1.84,-0.01)[c]	-0.46 (-1.23,0.31)
Medical Centre	-0.58 (-1.19,0.02)[d]	0.08 (-2.18,2.33)	-0.22 (-1.16,0.72)	0.12 (-0.82,1.06)	-0.30 (-1.19,0.59)	0.02 (-0.84,0.89)
Interaction with pharmacist only	-0.15 (-0.75,0.45)	-0.98 (-2.00,0.05)[d]	-0.27 (-0.45,-0.08)[b]	-0.23 (-0.50,0.04)[d]	-0.30 (-0.49,-0.11)[b]	-0.15 (-0.36,0.05)
**Interaction**						
Male staff # Male SP	[Reference]	[Reference]	[Reference]	[Reference]	[Reference]	[Reference]
Female staff # Female SP	0.64 (-1.39,2.67)	1.40 (0.53,2.28)[b]	0.36 (-1.32,2.03)	-0.29 (-1.84,1.26)	0.34 (-1.30,1.99)	-0.16 (-1.73,1.41)
Male staff # Female SP	NA	NA	0.20 (-0.54,0.94)	-0.21 (-0.92,0.49)	0.26 (-0.57,1.10)	0.09 (-0.53,0.72)
Female staff # Male SP	NA	NA	0.53 (-0.13,1.19)	-0.01 (-0.54,0.51)	0.48 (-0.18,1.14)	0.06 (-0.45,0.57)
N Questions Asked	0.16 (0.05,0.27)[b]	0.60 (0.20,0.99)[b]	-0.02 (-0.14,0.10)	0.10 (0.00,0.20)[c]	NA	NA
**N Diagnostic Questions Asked**						
No Diagnostic Question Asked	[Reference]	[Reference]	[Reference]	[Reference]	[Reference]	[Reference]
One Diagnostic Question	NA	NA	NA	NA	0.56 (0.11,1.01)[c]	0.65 (0.22,1.07)[b]
Both Diagnostic Questions	NA	NA	NA	NA	0.12 (-0.58,0.83)	0.14 (-0.93,1.21)
**Time of day**						
Morning (before 12pm)	-0.05 (-2.59,2.48)	-0.23 (-1.08,0.62)	0.09 (-0.88,1.06)	-0.77 (-1.53,-0.01)[c]	0.13 (-0.81,1.08)	-0.70 (-1.43,0.03)[d]
Afternoon (12pm to 4pm)	[Reference]	[Reference]	[Reference]	[Reference]	[Reference]	[Reference]
Evening (after 4pm)	-1.48 (-2.85,-0.11)[c]	-1.99 (-3.33,-0.66)[b]	-0.23 (-1.08,0.61)	-0.17 (-1.12,0.79)	-0.31 (-1.25,0.63)	-0.18 (-1.13,0.77)
**Day of week**						
Monday to Thursday	[Reference]	[Reference]	[Reference]	[Reference]	[Reference]	[Reference]
Friday to Sunday	0.88 (0.10,1.65)[c]	0.89 (-0.21,1.99)	0.29 (-0.32,0.89)	0.33 (-0.07,0.73)	0.34 (-0.36,1.03)	0.27 (-0.07,0.61)
Case 2B (Viral)	NA	NA	1.77 (1.01,2.52)[a]	2.09 (1.18,2.99)[a]	1.78 (1.09,2.48)[a]	1.88 (1.11,2.65)[a]
Observations	41	44	154	154	154	154
Clusters	7	7	7	7	7	7
Pseudo R2	0.352	0.573	0.323	0.381	0.339	0.387

[a] $P < .001$. [b] $P < .01$. [c] $P < .05$. [d] $P < .1$.

[e] Omitted because non-compliant behavior was observed in all three stand-alone pharmacies. Robust standard errors were clustered on retail group levels.

© 2019 Smith H et al. *JAMA Network Open.*

eFigure. Distribution of Pharmacies Visited
Regional boundaries are from the Australian Bureau of Statistics' 2016 Statistical Area Level 2 (SA2). The colors indicate the number of pharmacies visited within each region, by scenario.

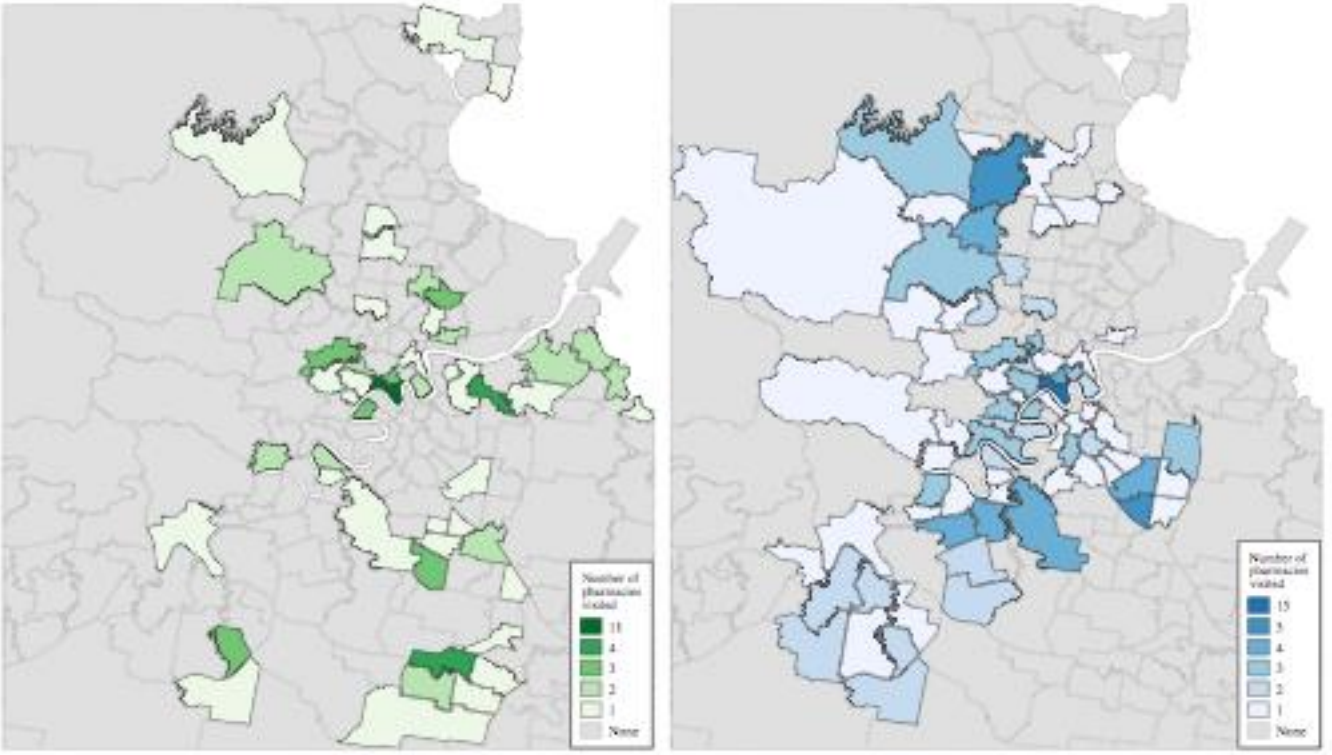

© 2019 Smith H et al. *JAMA Network Open.*

eAppendix. Research Assistant Checklist

Interaction Details

What is the exact time of day?
[input text]

Who did you interact with in the pharmacy?
[select one]
Pharmacist Only
Pharmacist Assistant Only
Pharmacist Assistant – then referred to Pharmacist
Pharmacist Assistant – consulted with Pharmacist for Advice
Unsure – Staff member did not identify themselves/no name badge

The person I interacted with was:
[select one]
Male
Female

Estimated age of the person I interacted with was:
[select one]
Under 20
21-30
31-40
41-50
51-60
60+

Estimated time of interaction in minutes:
[select one]
Less than 1 minute
1 mminute
2 minutes
3 minutes
4 minutes
5 minutes
6 minutes
7 minutes
8 minutes
9 minutes
10 minutes
11 minutes
12 minutes
13 minutes
14 minutes
15 minutes
More than 15 minutes

Please enter the NAME of the treatment(s) or medication(s) were you prescribed/sold:
If nothing, write "nothing"
[input text]

© 2019 Smith H et al. *JAMA Network Open.*

Please enter the PRICE of the treatment or medication you were prescribed/sold (in dollars):
If nothing, write "nothing"
[input text]

Were you asked if you would like the "generic" medication?
Eg. Cheaper pharmacist brand medication/product
[select one]
Yes
No
Not applicable

Were you asked if you were taking any other medication?
[select one]
Yes
No
Not applicable

Were you asked if you had any medical conditions?
[select one]
Yes
No
Not applicable

Were you asked if you have any allergies or have had a bad reaction to medication in the past?
[select one]
Yes
No
Not applicable

Was the recommended treatment option(s) discussed with you?
[select one]
Yes
No
Not applicable

Were you given advice on the recommended treatments use/dosage?
[select one]
Yes
No
Not applicable – referred to doctor without supplying anything

Were you given advice on how long you should take the medication for?
[select one]
Yes
No
Not applicable – referred to doctor without supplying anything

Were you given advice on treatment expectations?
Eg. When it should start working
[select one]

© 2019 Smith H et al. *JAMA Network Open.*

Yes
No
Not applicable – referred to doctor without supplying anything

Were negative effects of medication/treatment discussed with you?
[select one]
Yes
No
Not applicable – referred to doctor without supplying anything

Were you provided with any information about lifestyle modification?
[select one]
Yes
No
Not applicable

Were you offered any follow up advice?
Eg. If symptoms persist see doctor, or consumer medical pamphlet
[select one]
Yes
No
Not applicable

Was there anything else about the interaction that you think should be recorded for the researchers?
[input text]

About the Pharmacy

Pharmacy location:
[select one]
Shopping centre
Stand alone
Medical centre
Strip
Other

Is the Pharmacy a franchise group?
[select one]
Yes
No

Does the Pharmacy advertise a price match policy?
[select one]
Yes
No

© 2019 Smith H et al. *JAMA Network Open.*
